# Low Cycle Fatigue Life Prediction for Hydrogen-Charged HRB400 Steel Based on CPFEM

**DOI:** 10.3390/ma18163920

**Published:** 2025-08-21

**Authors:** Bin Zeng, Xue-Fei Wei, Ji-Zuan Tan, Ke-Shi Zhang

**Affiliations:** 1 School of Civil Engineering and Transportation, Foshan University, Foshan 528225, China; zengbin@fosu.edu.cn; 2 School of Mechatronic Engineering and Automation, Foshan University, Foshan 528225, China; 3 School of Civil Engineering and Architecture, Baise University, Baise 533000, China; tanedupro@163.com; 4 Key Lab of Disaster Prevention and Structural Safety, School of Civil Engineering and Architecture, Guangxi University, Nanning 530004, China

**Keywords:** hydrogen-induced fatigue damage, low-cycle fatigue, hydrogen embrittlement index, fatigue life prediction, microscale deformation inhomogeneity, crystal plasticity finite element

## Abstract

Addressing the limitations of traditional fatigue life prediction methods, which rely on extensive experimental data and incur high costs, and given the current absence of studies that employ deformation inhomogeneity parameters to construct fatigue-indicator parameter (FIP) for predicting low-cycle fatigue (LCF) life of metals in hydrogen environments, this study firstly explores how hydrogen pre-charging influences the LCF behavior of hot-rolled ribbed bar grade 400 (HRB400) steel via experimental and crystal plasticity simulation, and focus on the relationship between the fatigue life and the evolution of microscale deformation inhomogeneity. The experimental results indicate that hydrogen charging causes alterations in cyclic hysteresis, an expansion of the elastic range of the stabilized hysteresis loop, and a significant reduction in LCF life. Secondly, a novel FIP was developed within the crystal plasticity finite element method (CPFEM) framework to predict the LCF life of HRB400 steel under hydrogen influence. This FIP incorporates three internal variables: hydrogen embrittlement index, axial strain variation coefficient, and macroscopic stress ratio. These variables collectively account for the hydrogen charging effects and stress peak impacts on the microscale deformation inhomogeneity. The LCF life of hydrogen-charged HRB400 steel can be predicted using this new FIP. We performed fatigue testing under only one loading condition to measure the corresponding fatigue life and determine the FIP critical value. This helped predict fatigue life under different cyclic loading conditions for the same hydrogen-charged material. We compared the experimental data to validate the novel FIP to accurately predict the LCF life of hydrogen-charged HRB400 steel. The error between the predicted results and the measured results is limited to a factor of two.

## 1. Introduction

HRB400 steel is a widely used reinforcement material in concrete structures, particularly in marine engineering applications, such as cross-sea bridges and underwater tunnels, owing to its good mechanical properties and the relatively low cost. However, the HRB400 steel components face complex conditions in marine environments, and experience high stress and significant plastic deformation due to low-cycle fatigue (LCF) loading, along with severe challenges due to hydrogen corrosion (e.g., seawater electrolysis) [1,2,3]. Hydrogen atoms diffuse and accumulate within the material under cyclic loading, which potentially induces hydrogen-induced fatigue fracture and causes catastrophic failures [4,5,6]. Therefore, the accurate prediction of the fatigue life of HRB400 steel under hydrogen influence is crucial for implementing preventive measures and ensuring the safe and stable operation of these structures.

The fatigue life prediction models for metallic materials can be categorized into the macroscopic and mesoscopic approaches. Macroscopic phenomenological models have been extensively applied in various fields such as hydrogen transmission pipelines and nuclear systems [7,8,9]. However, these models typically require extensive fatigue testing data and lack a direct link to microscale damage mechanisms, thereby limiting their ability to elucidate the underlying physics of fatigue failure. On the mesoscopic level, the crystal plasticity finite element method (CPFEM) combines the crystal plasticity theoretical model with the finite element method (FEM). This method exhibits the slip-dominated deformation mechanism at the mesoscale and effectively captures the material’s microscopic evolution characteristics. These include the fatigue indicator parameter (FIP), which is crucial for predicting the fatigue life of metallic materials. Various FIPs were proposed based on the cumulative plastic slip [10,11,12], strain energy dissipation [13,14,15], and mesoscale deformation inhomogeneity [16,17] to address various application scenarios. A life prediction methodology was developed in [18] based on the mesoscale deformation inhomogeneity. This approach enabled the accurate prediction of tension-compression strain-controlled fatigue life for GH4169 superalloy by determining the FIP critical value through fatigue testing at a single specified strain amplitude. This methodology was extended to simulate torsional fatigue cycling of 30CrMnSiNi2A steel, where the predicted fatigue life fell within an acceptable error range corresponding to experimental measurements [19].

However, predicting the fatigue life of hydrogen-charged metals remains a major challenge. These difficulties are attributed to the interaction between hydrogen and the microstructure of the material under applied loads, coupled with hydrogen-promoted multiple failure mechanisms and the difficulty in determining damage parameters [20,21,22]. Hydrogen, being the smallest element, diffuses readily through grain boundaries or voids and migrates rapidly within the material. This diffusion promotes hydrogen embrittlement in metals, ultimately leading to fatigue failure [23]. Given the experimental challenges in directly observing the dynamic behavior of hydrogen atoms, efforts have been made to elucidate the physical mechanisms of hydrogen-induced failure by investigating changes in the material’s mechanical behavior and the microscopic morphology at failure sites [24,25,26]. For example, Komatsu et al. [24] investigated the formation mechanisms of hydrogen-induced vacancies in 316L stainless steel under varying strains and temperatures, demonstrating that localized strain regions constitute critical initiation points for embrittlement. Wang et al. [25] analyzed the influence of hydrogen on the fatigue fracture microstructure of a ferrite–pearlite dual-phase low-carbon steel, concluding that the hydrogen-enhanced localized plasticity (HELP) theory provides a robust explanation for the observed dislocation structure evolution in the fatigue fracture zone. Furthermore, numerous researchers have assessed the fatigue life and performance of smooth and notched specimens under hydrogen influence [27]. For instance, Hwang et al. [28] investigated the hydrogen embrittlement behavior of notched 316L stainless steel subjected to in situ electrochemical hydrogen charging. Their work demonstrated that localized hydrogen accumulation induced by the notch, combined with stress concentration, acts as a critical factor promoting brittle crack initiation.

The theoretical frameworks required for predicting the fatigue life of hydrogen-containing metals are still nascent. Most previous studies relied on specific tests to analyze the impact of hydrogen on the fatigue life, and limited research was conducted on employing micromechanics-based approaches. Recently, machine learning (ML) techniques have emerged as successful tools for predicting the fatigue properties of metallic materials [29,30,31], including hydrogen-containing metals. However, these approaches are considerably hindered by the requirement for extensive experimental data. Conversely, the fatigue prediction approach employs deformation inhomogeneity characterization parameters as the FIP, and presents distinct advantages. This method requires only a single fatigue test at a specified strain amplitude to determine the FIP critical value. Once established, this critical value helps accurately predict fatigue life under other strain amplitudes. When compared with other FIP-based methods and ML approaches, this method is easier to implement and provides life predictions prior to extensive fatigue testing. However, no relevant studies have been reported on the implementation of this specific method to predict the LCF life of metals under the influence of hydrogen.

In summary, although the FIP based on mesoscale deformation inhomogeneity achieves relatively accurate predictions for the LCF life of metallic materials within the CPFE framework, limited research has been conducted on its application for the prediction of the LCF life under hydrogen influence. This study proposes modifications to the existing mesoscale deformation inhomogeneity-based FIP. The modified FIP presents a model that can accurately predict the LCF life of uncharged and hydrogen-pre-charged HRB400 steel. First, the impact of hydrogen pre-charging on the LCF behavior of HRB400 steel was investigated through experimental testing and crystal plasticity numerical simulations. Concurrently, we analyzed the correlation between the fatigue life and mesoscale deformation inhomogeneity. Subsequently, a novel statistical characteristic parameter within the CPFE framework was proposed. Distinct from the existing strain statistical parameters, this new parameter incorporates the hydrogen embrittlement index, the coefficient of variation for axial strain, and the macroscopic stress ratio as the internal state variables. Finally, this new parameter was employed as the FIP to predict the LCF life curve of the hydrogen-pre-charged HRB400 steel, and its predictive capability was validated through experimental measurements.

This study aims to propose a novel FIP for predicting the LCF life of HRB400 steel under hydrogen influence. The main contributions of this work can be summarized as follows. (1) To investigate the effect of pre-charged hydrogen on the LCF behavior of HRB400 steel through experimental testing and crystal plasticity numerical simulations, and to explore the relationship between fatigue life and the evolution of microscale deformation inhomogeneity. (2) Aiming to predict the LCF life of HRB400 steel under hydrogen influence, a new FIP accounting for hydrogen effects is proposed by introducing the hydrogen embrittlement index, the axial strain variation coefficient, and the macroscopic stress ratio as internal variables.

## 2. LCF Testing of Hydrogen-Charged HRB400 Steel

### 2.1. Materials and Testing Methods

**Figure 1:**
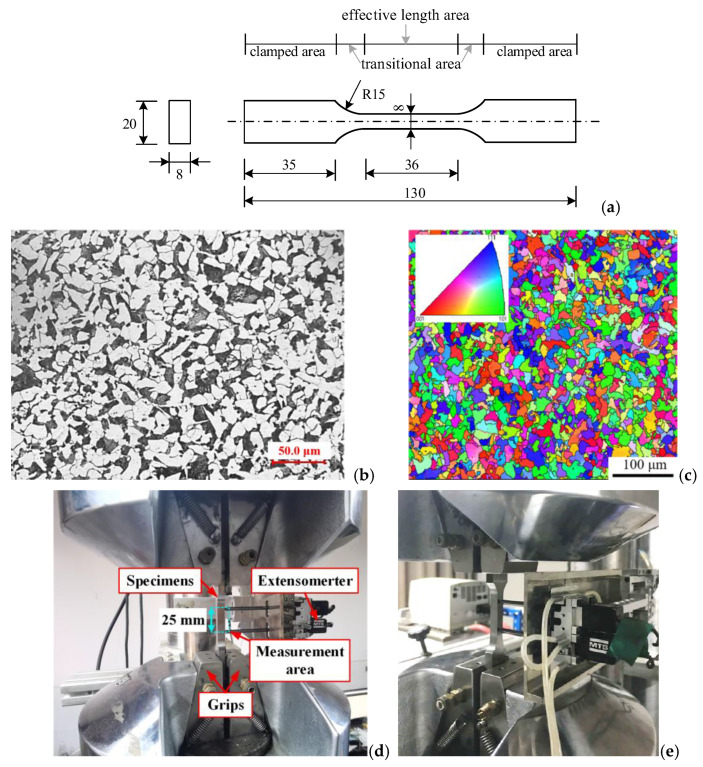
Test sample and experimental equipment: (**a**) sample geometry (unit: mm) [32]; (**b**) sample metallographic structure; (**c**) sample microstructure and grain orientation; and (**d**,**e**) loading and measuring equipment.

The experimental material, HRB400 steel, was produced via conventional hot rolling by Guangxi Guigang Iron and Steel Group Co., Ltd., Guigang, China. Its chemical composition (wt%) as certified by the manufacturer is: C 0.23, Mn 1.3, Si 0.37, P 0.032, S 0.015, Ceq 0.48, Fe rest. Plate samples were fabricated by initial rough machining on a conventional lathe, followed by precision finishing using a computer numerical control (CNC) lathe and subsequent grinding. All specimens underwent final polishing. The sample geometry comprises three distinct regions: a clamping area, transition area, and effective length area, as schematically illustrated in Figure 1a [32]. The metallographic structure of HRB400 steel, as well as the microstructure and grain orientation measured by EBSD, are shown in Figure 1b,c.

**Figure 2:**
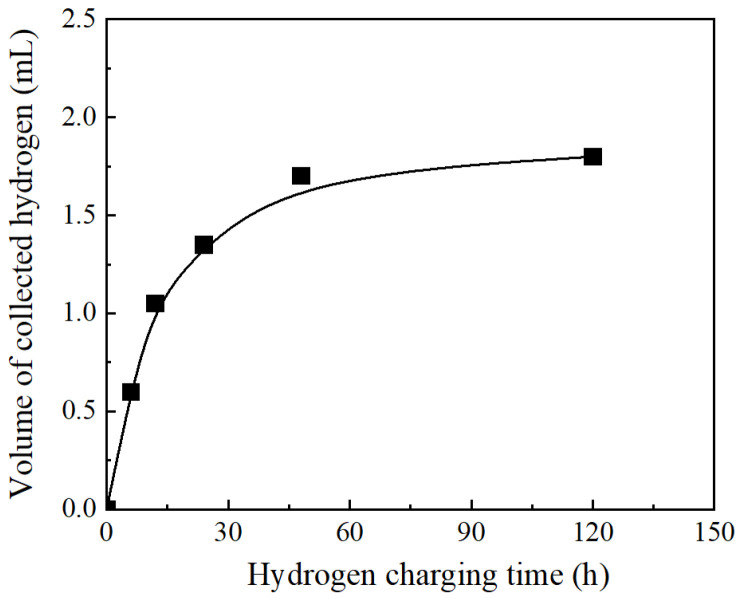
Variation of hydrogen content inside the specimen with hydrogen charging time.

Hydrogen charging of the samples was conducted via the cathodic hydrogen charging technique, employing an electrochemical cell configuration. The electrolytic solution consisted of sulfuric acid (H_2_SO_4_) dissolved in distilled water. samples served as the cathode, while three carbon rods (Ф6 × 100 mm), distributed uniformly around the sample, served as the anode. Samples were categorized into three groups according to differing hydrogen charging conditions, detailed in Table 1 [32]. Hydrogen content was measured using the glycerol displacement method to estimate internal hydrogen concentration in hydrogen-charged specimens. A collection period of 30 days was employed to ensure complete release of hydrogen from the steel. The resulting curve depicting internal hydrogen concentration versus charging time is presented in Figure 2.

To investigate the influence of hydrogen on the LCF characteristics of HRB400 steel, symmetric strain-controlled fatigue experiments were conducted following hydrogen charging. Experiments were conducted on an MTS809 testing machine (shown in Figure 1d,e), employing an MTS axial extensometer mounted at the mid-span of the sample’s effective length. The extensometer, configured with a 25 mm gauge length, and the ultimate axial strain was 10%. Five test groups were conducted, with strain amplitudes of 0.35%, 0.4%, 0.6%, 0.8%, and 1.0%, respectively. Strain-controlled testing was implemented via a 1 Hz triangular waveform loading pattern.

It should be noted that the mechanical properties of hydrogen-charged HRB400 steel obtained from tensile tests have been previously published by the authors in [32]. It is observed that HRB400 steel exhibits a distinct yield plateau, and hydrogen charging alters its yield strength. In the cyclic tests conducted in this study, the deformation type for all specimens was elastoplastic cyclic deformation.

### 2.2. Low Cycle Fatigue Response

#### 2.2.1. Cyclic Stress Response

**Figure 3:**
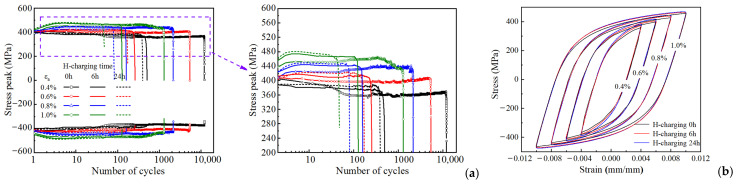
Cyclic response of HRB400 steel samples under varying strain amplitudes and hydrogen charging durations: (**a**) cyclic stress response curves; (**b**) cyclic stabilization hysteresis loops.

The cyclic stress response curve serves as a critical metric for cyclic deformation behavior. As shown in Figure 3a, the evolution of peak and valley stresses versus cycle number is presented for hydrogen-charged HRB400 steel specimens across various strain amplitudes (for clarity, the curve corresponding to the 0.35% strain amplitude is excluded from the figure, as its peak stress exhibits minimal deviation from that observed at 0.4%). The results demonstrate fundamentally stable cyclic stress response. Notably, hydrogen charging modifies the material’s initial cyclic softening/hardening characteristics during early loading stages. Distinctly different phenomena were observed under identical hydrogen charging conditions. At strain amplitudes below 0.6%, the peak stress decreases with an increase in the cycle number (softening), whereas at amplitudes exceeding 0.6%, it increases (hardening). Upon reaching cyclic stability, the peak stress consistently exhibits a slight decrease with further cycling, thereby indicating a weak softening tendency. Furthermore, the hydrogen charging duration significantly influences the cyclic peak stress magnitude, thereby exhibiting a positive correlation where longer charging times yield a higher peak. HRB400 steel exhibits a distinct yield plateau. This plateau effect is a key factor in the different softening/hardening behaviors observed under cyclic loading at various strain amplitudes. The impact of this effect on cyclic hardening and softening behaviors is most pronounced during the initial loading cycles but progressively weakens with increasing cycle number.

#### 2.2.2. Analysis of Hysteresis Loop

As cyclic loading progresses, the hysteresis curves for all the amplitudes evolve towards a stable state, thereby forming stabilized hysteresis loops. As presented in Figure 3b, stabilized hysteresis loops at mid-life (corresponding to half the fatigue life) are compared across different strain amplitudes. It is evident that hydrogen charging significantly modifies the stress range of stabilized hysteresis loops, with extended charging durations further elevating their stress amplitude.

**Figure 4:**
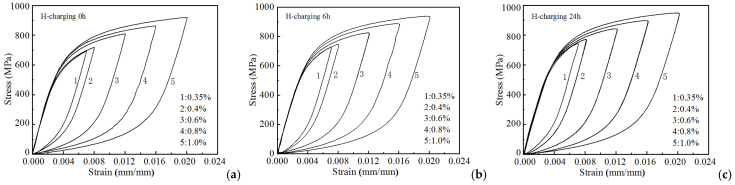
Translate the lowest point of the cycle stability hysteresis loops of the sample with different hydrogen charging time to the coordinate origin (0,0): (**a**) 0 h, (**b**) 6 h, (**c**) 24 h.

Figure 4 depicts the stabilized hysteresis loops obtained by translating the lower vertices of the loops presented in Figure 4 to the coordinate origin. Moreover, curves 1 and 2 in the figure are nearly coincident, whereas the remaining curves do not overlap. This indicates that the material exhibits Masing behavior under low strain amplitude conditions, whereas it exhibits non-Masing behavior under high strain amplitude conditions. Each hysteresis loop was subsequently translated along the elastic line and aligned at the yield point. The result shows that the strain hardening curves for each hysteresis loop exhibit essential coincidence, thus enabling master curve construction [32]. Figure 4a illustrates this master curve derivation for non-hydrogen-charged specimens. Moreover, the master curve equation corresponding to the smallest strain amplitude hysteresis loop is given by:

(1) $$\varepsilon^{*}=\frac{\sigma^{*}}{E}+2{\left(\frac{\sigma^{*}}{2K^{*}}\right)}^{1/n^{*}},$$

where the superscript asterisk (*) designates experimental data referenced to the transformed coordinate system. Parameters $K^{*}$ and n* were determined through curve fitting of Equation (1) to experimental results, yielding values of $K^{*}$ = 810 MPa and n* = 0.134.

**Figure 5:**
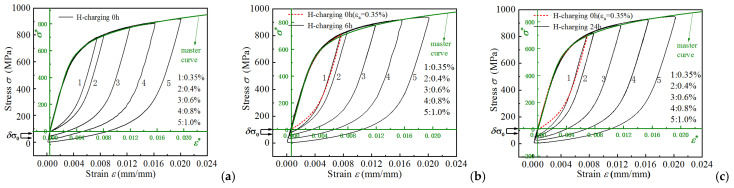
Coincidence in the loading segments of stabilized hysteresis loops for different hydrogen charging times: (**a**) 0 h, (**b**) 6 h, (**c**) 24 h.

Figure 5b,c contrast stabilized hysteresis loops of 6 h/24 h hydrogen-charged specimens under different cyclic loading conditions with the Equation (1) master curve. Non-hydrogen-charged sample reference loops (0.35% strain amplitude) are shown in red. The critical parameter $\delta \sigma_{0}$, defined as the loading branch-to-master curve deviation, measures divergence from Masing material response. This metric directly reflects proportional limit stress enhancement (or elastic range, and $\delta \sigma_{0}=0$ denoting Masing material), governed by:

(2) $$\delta \sigma_{0}=\sigma -\sigma^{*}=\sigma -2K^{*}{\left(\frac{\varepsilon_{P}}{2}\right)}^{n^{*}}.$$

**Figure 6:**
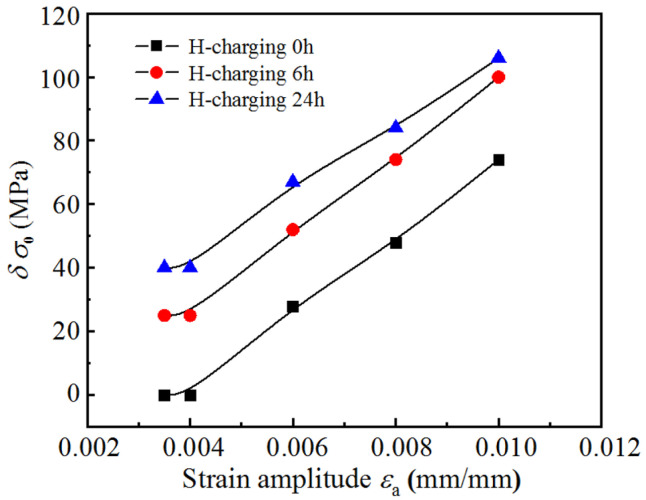
Relationship between the increase in the proportional stress limit, *δσ*_0_, and the strain amplitude.

The magnitude of $\delta \sigma_{0}$ quantified the impact of strain amplitude and hydrogen charging duration on the stabilized hysteresis loops. Figure 6 depicts the relationship between the elastic range, $\delta \sigma_{0}$, and strain amplitude for three sets of hydrogen-charged samples. The $\delta \sigma_{0}$ for stabilized loops increases with strain amplitude. Moreover, hydrogen charging extends the elastic range of these loops; this effect becomes more pronounced with longer hydrogen charging durations.

#### 2.2.3. Strain-Life Relationships

**Figure 7:**
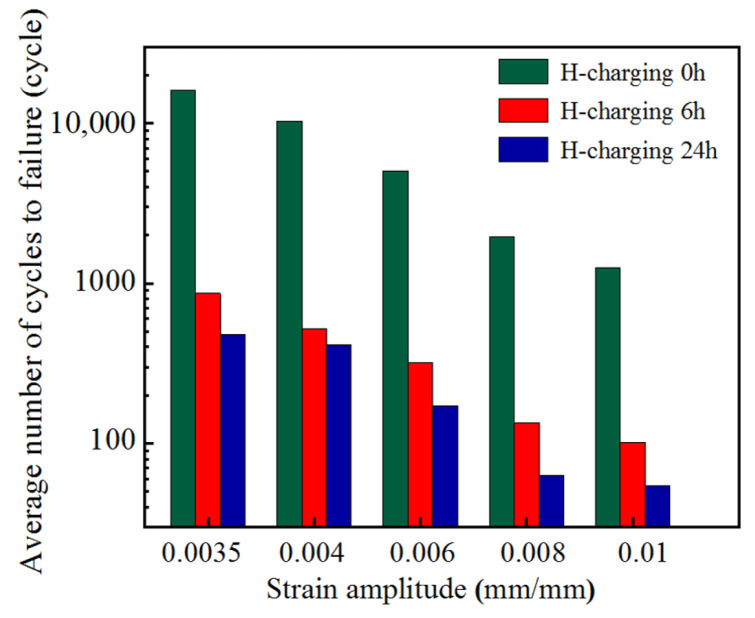
Average fatigue life of three groups of HRB400 steel samples under different strain amplitudes.

For all the tests, loading was terminated upon the observation of a distinct load drop and surface crack initiation on the specimens. The fatigue life was defined as the corresponding cycle count at failure. Figure 7 presents the average fatigue life of hydrogen-charged samples under various strain amplitudes. Key observations include the following: ① For identical hydrogen charging durations, higher strain amplitudes reduced fatigue life. ② Under identical loading conditions, hydrogen-charged samples exhibited approximately one order of magnitude shorter fatigue lives compared to uncharged counterparts, with life reduction severity escalating with prolonged hydrogen charging. Based on Figure 2, it is evident that within the hydrogen charging time range not exceeding 24 h, the hydrogen content in the specimens increases with charging time. This demonstrates that the low-cycle fatigue life of HRB400 steel decreases with increasing hydrogen content. It can be seen that LCF life is related to strain amplitude and hydrogen content. As the strain amplitude increases, the area of the hysteresis loop expands, resulting in higher energy dissipation. This accelerates crack propagation. Furthermore, during repeated loading, hydrogen further accelerates both the formation of fatigue origins and the propagation of fatigue cracks.

Subsequently, the effect of hydrogen charging duration on LCF was evaluated via the strain amplitude-life relationship. In strain-controlled cycling, this correlation is described by the Morrow equation:

(3) $$\varepsilon_{a}=\varepsilon_{e}+\varepsilon_{p}=\frac{\sigma_{f}}{E}{\left(2N_{f}\right)}^{b}+\varepsilon_{f}{\left(2N_{f}\right)}^{c},$$

here, $\varepsilon_{e}$, $\varepsilon_{p}$, and $\varepsilon_{a}$ denote the elastic, plastic, and total strain amplitudes, respectively. 2*N_f_* denotes the number of reversals to failure (where *N_f_* denotes the fatigue life value), and *E* is the material’s elastic modulus. Material parameters *b* and $\sigma_{f}$ represent the fatigue strength exponent and coefficient, respectively. *c* and $\varepsilon_{f}$ represent the fatigue ductility exponent and coefficient, respectively.

**Figure 8:**
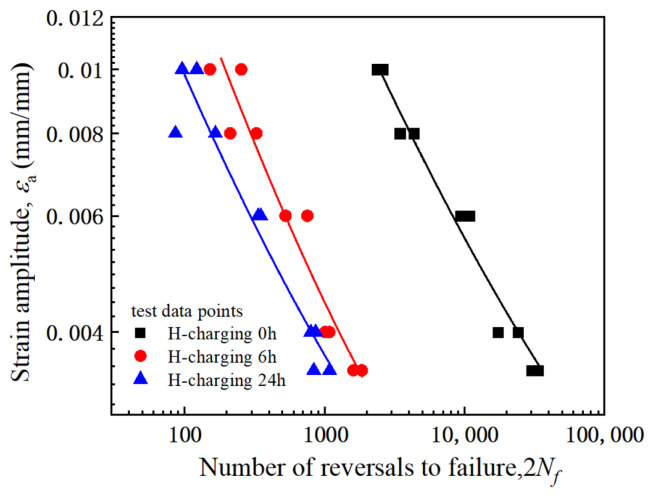
2*N_f_*-$\varepsilon_{a}$ curves of HRB400 steel samples with different hydrogen charging times in double logarithmic coordinates.

The experimentally measured fatigue life data points for three sets of hydrogen-charged samples were plotted on double logarithmic coordinates and fitted using Equation (3), thereby yielding the results presented in Figure 8. Furthermore, Table 2 lists the fatigue ductility and strength parameters derived from the data fitting procedure. Hydrogen charging alters the fatigue strength parameters and fatigue ductility parameters.

## 3. Theory of Crystal Plastic Finite Element Method (CPFEM)

### 3.1. Crystal Plasticity Constitutive Model

Within the crystal plasticity framework, the Euler rate gradient tensor, **L**, at material points admit the decomposition into the elongation tensor, **D**, and elongation tensor, W [33,34,35]:

(4) $$D=D^{*}+D^{P}=\mathrm{sym}\left(L^{*}\right)+\mathrm{sym}\left(L^{P}\right),\;W=W^{*}+W^{p}=\mathrm{asym}\left(L^{*}\right)+\mathrm{asym}\left(L^{P}\right),$$

where $D^{*}$ and $D^{P}$ denote the elastic and plastic elongation tensor, and $W^{*}$ and $W^{p}$ represent the elastic and plastic spin tensors, respectively.

For small elastic deformations, the rate constitutive relation takes the form:

(5) $${\dot{\sigma }}^{J}=\overset{\langle 4\rangle }{C}:D^{*}=\overset{\langle 4\rangle }{C}:\left(D-D^{P}\right),$$

where $\overset{\langle 4\rangle }{C}$ denotes the fourth-order elastic constitutive tensor of the crystal in a fixed global coordinate system. The Jaumann rate of the Cauchy stress, denoted ${\dot{\sigma }}^{J}$, is defined as [18]:

(6) $${\dot{\sigma }}^{J}=\dot{\sigma }-W\cdot \sigma +\sigma \cdot W,$$

where σ and $\dot{\sigma }$ represent the Cauchy stress tensor and its rate, respectively. The logarithmic strain increment, Δε, is obtained by time-integrating the elongation tensor, D, from *t* to *t* + ∆*t*. The incremental accumulation of σ can be updated via Equations (5) and (6):

(7) σt+Δt−σtt+Δt=ΔσJ, or σt+Δt=σtt+Δt+C〈4〉:Δε−Δεp,

where $\Delta \varepsilon^{p}$ is the logarithmic plastic strain increment. σtt+Δt and σt+Δt are the Cauchy stresses corresponding to times *t* and *t* + ∆*t*, respectively.

The correlation between shear stress and shear strain rate in the alpha-slip system is described by incorporating the concept of back stresses, as presented in [36,37].

(8) $${\dot{\gamma }}^{\left(\alpha \right)}={\dot{\gamma }}_{0}\mathrm{sgn}\left(\tau^{\left(\alpha \right)}-x^{\left(\alpha \right)}\right){\left|\frac{\tau^{\left(\alpha \right)}-x^{\left(\alpha \right)}}{g^{\left(\alpha \right)}}\right|}^{k},$$

where $\tau^{\left(\alpha \right)}$ and $x^{\left(\alpha \right)}$ represent the decomposed shear stress and its associated back stress for the *α*th slip system in a single crystal, respectively. $g^{\left(\alpha \right)}$ is a scalar function that defines the elastic limit of the shear stress within the *α*th slip system. ${\dot{\gamma }}_{0}$ denotes the reference shear strain rate constant, while *k* is the rate-dependent parameter. The development of the back stress, $x^{\left(\alpha \right)}$, is further modeled using an improved version of the Armstrong–Frederick (A-F) model, which is expressed as follows [38,39]:

(9) $${\dot{x}}^{\left(\alpha \right)}=a{\dot{\gamma }}^{\left(\alpha \right)}-c\left[1-e_{1}\left(1-\exp\left(-e_{2}\gamma \right)\right)\right]x^{\left(\alpha \right)}\left|{\dot{\gamma }}^{\left(\alpha \right)}\right|-px^{\left(\alpha \right)},$$

where a denotes the linear hardening of the slip system. Parameters c and p define the characteristics of nonlinear kinematic hardening, whereas $e_{1}$ and $e_{2}$ correspond to the rates for the nonlinear decay and saturation of back-stress under cumulative shear strain. These five parameters can be derived from crystal plasticity numerical simulations of the RVE model by utilizing experimental data on hysteresis behavior from cyclic tests.

The evolution of the scalar function, $g^{\left(\alpha \right)}$, in Equation (8), is governed by [40]:

(10) $${\dot{g}}^{\left(\alpha \right)}(\gamma )=\sum_{\beta =1}^{n}h_{\alpha \beta }(\gamma )\left|{\dot{\gamma }}^{\left(\beta \right)}\right|,\gamma =\int \sum_{\beta =1}^{n}\left|d\gamma^{\left(\beta \right)}\right|,$$

where $h_{\alpha \beta }$ is the slip hardening modulus, which Hutchinson [41] formulated as:

(11) $$h_{\alpha \beta }(\gamma )=h(\gamma )\left[q+\left(1-q\right)\delta_{\alpha \beta }\right],$$

where q is a constant, and h(γ) can be calculated as follows [42]:

(12) $$h(\gamma )=h_{0}{\mathrm{sech}}^{2}\left(\frac{h_{0}\gamma }{\tau_{s}-\tau_{0}}\right),$$

where $h_{0}$ is the initial hardening rate, while $\tau_{0}$ and $\tau_{s}$ represent the critical shear stress at initial state and saturated state, respectively. These material constants are calibrated through integrated experimental–numerical analysis.

For the *α*th slip system, $m^{\left(\alpha \right)}$ (the unit vector in the initial slip direction) and $n^{\left(\alpha \right)}$ (the unit vector in the initial slip plane normal) define the Schmid tensor in the global Cartesian frame [34,43]:

(13) $$\begin{array}{l} P^{(\alpha )*}=\frac{1}{2}\left(m^{(\alpha )*}n^{(\alpha )*}+n^{(\alpha )*}m^{(\alpha )*}\right),\;\\ \mathrm{and}\;m^{(\alpha )*}=F^{*}\cdot m^{\left(\alpha \right)},n^{(\alpha )*}=n^{\left(\alpha \right)}\cdot F^{*-1}, \end{array}$$

where $F^{*}$ is the elastic part of the deformation gradient tensor.

In Equation (7), the increment of the logarithmic strain tensor is determined based on Schmid tensor, as outlined in [33]:

(14) $$\Delta \varepsilon^{p}=\sum_{\alpha =1}^{n}P^{(\alpha )*}\Delta \gamma^{\left(\alpha \right)},$$

where $\Delta \gamma^{\left(\alpha \right)}$ is obtained by integrating ${\dot{\gamma }}^{\left(\alpha \right)}$.

The decomposed shear stress, $\tau^{\left(\alpha \right)}$, in the *α*th slip system relates to Cauchy stress as:

(15) $$\tau^{\left(\alpha \right)}=P^{(\alpha )*}:\sigma .$$

The above model’s computational procedures can be implemented through the UMAT subroutine within the ABAQUS finite element platform [18,39].

### 3.2. RVE Model Formulation

**Figure 9:**
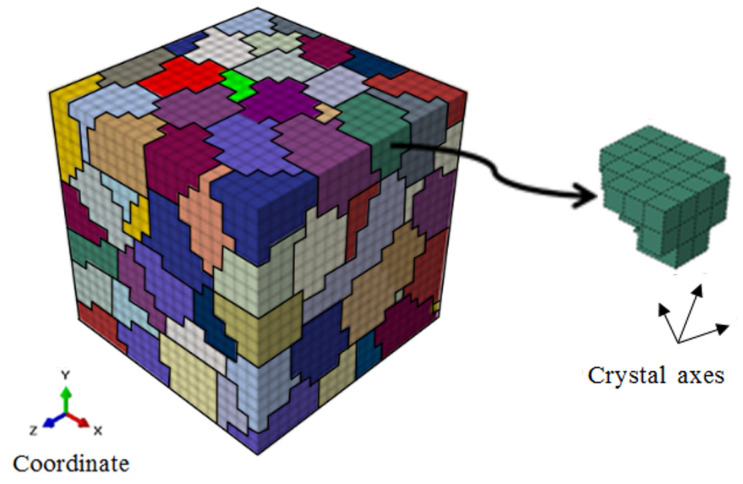
Polycrystalline RVE material model.

This study employs a polycrystalline representative volume element (RVE) model generated via the Voronoi method and integrated with crystal plasticity calculations, to simulate the macroscopic deformation and inhomogeneous mesoscopic deformation of HRB400 steel. The RVE comprises 8000 8-node hexahedral elements, with 125 grains exhibiting anisotropic mechanical behavior, as shown in Figure 9. The analysis specifically focuses on grain anisotropy and slip deformation mechanisms under cyclic loading, and excluding contributions from metallurgical defects, grain boundary interactions, and size effects.

The RVE model can successfully replicate both internal deformation inhomogeneity and macroscopic mechanical behavior observed in material testing. To closely replicate the material’s physical state, however, computational simulations require appropriate boundary constraints to be imposed on the RVE [44]. Under uniaxial tensile-compression cyclic loading, a simplified periodic boundary condition can be implemented by assuming planar preservation of RVE surfaces [17]. In particular, the model is configured such that displacement (or positive stress) along the normal is specified on the positive surface orthogonal to the X-axis. The corresponding normal displacement is zero on the negative surface orthogonal to the X-axis, Y-axis, and Z-axis. The positive surface perpendicular to the X-axis and the Y-axis remains unforced and maintains a constant normal direction. This boundary setup guarantees consistency between the RVE’s macroscopic mechanical behavior with the specimen’s working section mechanical response under uniaxial tensile-compressive cyclic loading.

### 3.3. FIP Based on Microscale Deformation Inhomogeneity

Deformation inhomogeneity is quantified through statistical metrics, where the statistical strain mean, ${\bar{\varepsilon }}_{ij}$, within the RVE characterizes the extent of macroscopic deformation during cyclic loading. Concurrently, variations in the statistical strain standard deviation, ${\hat{\varepsilon }}_{ij}$, reflect the evolving inhomogeneity at the grain scale. These two parameters are calculated as [39]:

(16) $${\bar{\varepsilon }}_{ij}=\sum_{k=1}^{n_{\mathrm{RVE}}}{\left(\varepsilon_{ij}\right)}_{k}v_{k},{\hat{\varepsilon }}_{ij}=\sqrt{\sum_{k=1}^{n_{\mathrm{RVE}}}{\left(\varepsilon_{ij}\right)}_{k}^{2}v_{k}-{\bar{\varepsilon }}_{ij}^{2}},$$

where $n_{RVE}$ is the total finite element count in the RVE, ${\left(\varepsilon_{ij}\right)}_{k}$ denotes the logarithmic strain tensor component of the *k*th element. And $v_{k}$ represents the volume fraction of the *k*th element, and it is computed by:

(17) $$v_{k}=\Delta V_{k}/V,$$

where Δ*V_k_* is the volume of the *k*th element and *V* is the total volume of the RVE.

During low-cycle fatigue testing, cyclic loading was applied along the longitudinal direction (*l*-direction). The longitudinal strain components, $\varepsilon_{l\underline{l}}$(the underscore denotes no summation over index of *l*), of RVE were analyzed statistically. Their standard deviation, ${\hat{\varepsilon }}_{l\underline{l}}$, quantifies the degree of mesoscopic deformation inhomogeneity within the material. This parameter represents the evolution of the microstructure of the material, and this evolution persists until the material fails. The degree of heterogeneous mesoscopic deformation reaches a constant value (or critical value) with an increase in the number of cycles. This critical value is independent of the loading conditions for a given material at a specified temperature. The fatigue life can be established accordingly after determining the critical value of heterogeneous mesoscopic deformation (${\hat{\varepsilon }}_{l\underline{l},fatigue}$). According to [18,19,39], ${\hat{\varepsilon }}_{l\underline{l}}$ can be utilized as an FIP for predicting the material fatigue life under symmetric strain cycling conditions. The fatigue failure criterion can be expressed as follows:

(18) $${\hat{\varepsilon }}_{l\underline{l}}={\hat{\varepsilon }}_{l\underline{l},fatigue}.$$

### 3.4. FIP Considering Hydrogen Effects

Hydrogen typically exists in an atomic state in the low-carbon steel. Under stress, hydrogen atoms tend to accumulate along the stress fields of dislocations or combine with vacancies to form complexes. This leads to localized concentration of plastic strain, thereby accelerating the initiation and early-stage propagation of microcracks [45,46]. Under cyclic loading, the driving force for hydrogen diffusion is significantly enhanced. The reciprocating motion of dislocations and the cyclic variation in vacancy concentration promote continuous hydrogen enrichment at microdefects. This further enhances localized plastic deformation and significantly shortens low-cycle fatigue life [25]. In this regime, fatigue failure corresponds to both the microscale deformation inhomogeneity, along with the hydrogen charging time (hydrogen content) and the internal hydrogen distribution within the material. Considering these factors, we introduced the hydrogen embrittlement index (EI) and the coefficient of variation for axial strain (${\hat{\varepsilon }}_{l\underline{l}}$/${\bar{\varepsilon }}_{l\underline{l}}$) within the RVE to modify the parameter (${\hat{\varepsilon }}_{l\underline{l}}$) characterizing microscale deformation inhomogeneity, thereby yielding a new FIP that considers the hydrogen effects, which is defined as follows:

(19) $${\hat{\varepsilon }}_{l\underline{l},H}=\frac{1}{\sigma_{0}}[\sigma_{\max}]({\hat{\varepsilon }}_{l\underline{l}}+EI\frac{{\hat{\varepsilon }}_{l\underline{l}}}{{\bar{\varepsilon }}_{l\underline{l}}}),$$

where *σ*_0_ denotes the yield stress and *σ*_max_ denotes the stress peak of the macroscopic stable hysteresis loop. ${\hat{\varepsilon }}_{l\underline{l}}$ and ${\bar{\varepsilon }}_{l\underline{l}}$ denote the statistical standard deviation and mean value of the axial strain within the RVE, respectively. The hydrogen embrittlement index, EI, exhibits a monotonic relationship with the hydrogen charging duration (i.e., hydrogen concentration), thereby quantifying the hydrogen-induced material embrittlement or plasticity loss. ${\hat{\varepsilon }}_{l\underline{l}}$/${\bar{\varepsilon }}_{l\underline{l}}$ characterizes the relative dispersion of the microscale deformation (also known as the coefficient of variation). The product term of EI and ${\hat{\varepsilon }}_{l\underline{l}}$/${\bar{\varepsilon }}_{l\underline{l}}$ represents the additional impact of hydrogen on the microscale deformation inhomogeneity. The ratio, *σ*_max_/*σ*_0_, is considered a weighting factor for microscale deformation inhomogeneity, thereby representing the influence of the stress peak on the fatigue failure (the larger the macroscopic load peak, the more likely fatigue failure is to occur at that moment). The values of *σ*_0_ and EI are obtained from [32].

${\hat{\varepsilon }}_{l\underline{l},H}$ is employed to predict the LCF life of HRB400 steel samples charged with hydrogen. Failure occurs when the FIP, ${\hat{\varepsilon }}_{l\underline{l},H}$, satisfies the following condition:

(20) $${\hat{\varepsilon }}_{l\underline{l},H}={\hat{\varepsilon }}_{l\underline{l},H-fatigue},$$

where ${\hat{\varepsilon }}_{l\underline{l},H-fatigue}$ denotes the critical value, corresponding to the value of ${\hat{\varepsilon }}_{l\underline{l},H}$ at fatigue failure under cyclic loading. The specific value of ${\hat{\varepsilon }}_{l\underline{l},H-fatigue}$ depends only on the pre-charging duration of the material, and is independent of the loading conditions. It can be determined based on the evolution curve of ${\hat{\varepsilon }}_{l\underline{l},H}$ and the experimentally measured fatigue life.

## 4. Results and Discussion

### 4.1. Calibration and Validation of Model Parameters

The calibration of the material parameters for the CPFE constitutive model can be performed based on the methodology presented in [17]. In particular, the same strain value as that measured experimentally is applied to the RVE, and the calibration is performed iteratively using a trial-and-error approach to achieve consistency between the simulated overall stress–strain stabilized hysteretic response of the RVE and the corresponding experimental results. For materials that exhibit Masing behavior, a single set of parameters calibrated based on the measured hysteresis loop at a specified strain amplitude can be used to simulate the cyclic processes at different strain amplitudes. However, for materials with pronounced non-Masing characteristics, significant errors are observed when employing a single set of parameters to simulate the cyclic processes at various strain amplitudes [19]. Therefore, distinct sets of parameters must be calibrated for different operating conditions.

**Figure 10:**
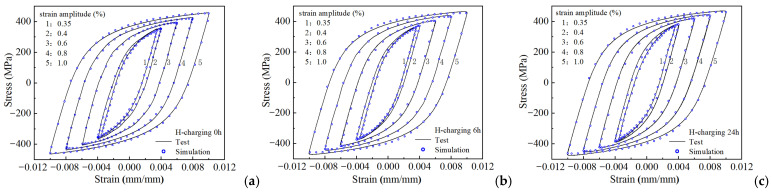
Experimental and simulated stabilized hysteresis loops for HRB400 steel at various strain amplitudes: (**a**) 0 h, (**b**) 6 h, (**c**) 24 h hydrogen charging.

As explained in Section 2.2, HRB400 steel exhibits non-Masing behavior. The deviation from the Masing characteristics manifests in the elastic range of the hysteresis loops, where the magnitude of this elastic range was influenced by hydrogen charging. Within the material parameters of the crystal plasticity constitutive model, the saturated value of the critical decomposed shear stress (*τ*_s_) helps in determining the simulated elastic range of the stabilized hysteresis curve. Consequently, *τ*_s_ requires separate calibration for distinct loading conditions. The specific procedure employed to calibrate the model parameters in this study is as follows. The stabilized hysteresis curve at a strain amplitude of 0.6% for the uncharged sample was employed to calibrate the model material parameters at this amplitude. Table 3 lists the resulting parameters. Subsequently, based on the material parameters listed in Table 3 (excluding *τ*_s_), the stabilized hysteresis loops of the uncharged, 6 h hydrogen-charged, and 24 h hydrogen-charged samples at different strain amplitudes are simulated to calibrate the corresponding *τ*_s_ values, as listed in Table 4. The cyclic loading processes of the samples with different hydrogen charging durations are simulated using the material parameters listed in Table 3 and Table 4. Figure 10 depicts a comparison between the simulated stabilized hysteresis loops and the experimentally measured curves. The results exhibit excellent concurrence between the simulated and experimental stabilized hysteresis curves.

Given the ambient temperature conditions and relatively low loading rates employed in the experiments, the parameter *k* necessitated a sufficiently large whereas ${\dot{\gamma }}_{0}$ required to be sufficiently small to reflect the low loading rates. Following Reference [17], the values *k* = 200 and ${\dot{\gamma }}_{0}$ = 0.01 were adopted. the ratio of latent hardening rate to self-hardening rate, *q*, typically ranging from 1.0 to 1.4, was assigned a value of *q* = 1 in this paper.

### 4.2. Determination of the FIP Critical Value

**Figure 11:**
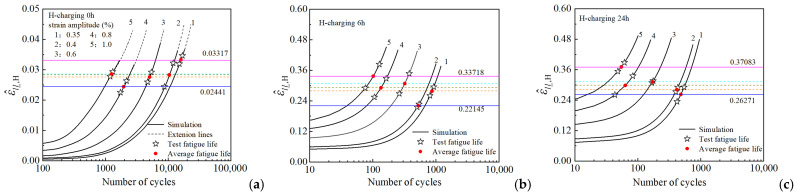
FIP (${\hat{\varepsilon }}_{l\underline{l},H}$) evolution with cycle number under strain-controlled cyclic loading for samples with different hydrogen charging times: (**a**) 0 h; (**b**) 6 h; (**c**) 24 h.

The fatigue life full-cycle processes of three groups of hydrogen-charged (0 h, 6 h, and 24 h) HRB400 samples under different loading conditions, based on the parameters calibrated in Section 4.1, were simulated. Consequently, the standard deviation (${\hat{\varepsilon }}_{l\underline{l}}$) and mean value (${\bar{\varepsilon }}_{l\underline{l}}$) of the axial strain within the RVE were obtained. Subsequently, the value of the FIP (${\hat{\varepsilon }}_{l\underline{l},H}$) at the cyclic tensile peak point was calculated using Equation (19). These three sets of curves represent the evolution of ${\hat{\varepsilon }}_{l\underline{l},H}$ with the number of cycles, respectively, as shown in Figure 11a–c. For each set of evolution curves, one representative curve was selected as the calibration curve to determine the FIP critical value, (${\hat{\varepsilon }}_{l\underline{l},H-fatigue}$). The fatigue life points obtained from the corresponding experiments, including individual test lifetimes (denoted by asterisks) and mean lifetimes (denoted by red dots), were plotted on these calibration curves. The value of ${\hat{\varepsilon }}_{l\underline{l},H}$ corresponding to the mean lifetime was determined as the critical value, ${\hat{\varepsilon }}_{l\underline{l},H-fatigue}$. Provided that the proposed fatigue failure criterion (Equation (20)) is valid and applicable, this critical value, ${\hat{\varepsilon }}_{l\underline{l},H-fatigue}$, can be employed to predict the fatigue lives of the samples under the same hydrogen charging conditions, but different loading amplitudes. The predicted fatigue life for a given loading condition is determined as the abscissa (number of cycles) of the intersection point between the horizontal line at ${\hat{\varepsilon }}_{l\underline{l},H-fatigue}$ and the corresponding ${\hat{\varepsilon }}_{l\underline{l},H}$ evolution curve.

For the uncharged sample (0 h), the critical values, (${\hat{\varepsilon }}_{l\underline{l},H-fatigue}$), obtained from the five evolution curves (each corresponding to a different loading condition) range from 0.02441 (minimum) to 0.03317 (maximum), with intermediate values of 0.02756, 0.02830, and 0.02854, as shown in Figure 11. Similarly, for the samples subjected to 6 h of hydrogen charging, the critical values range from 0.22145 (minimum) to 0.33718 (maximum), with intermediate values of 0.27895, 0.29219, and 0.30832. For the samples subjected to 24 h of hydrogen charging, the critical values range from 0.26271 (minimum) to 0.37083 (maximum), with intermediate values of 0.28098, 0.29864, and 0.31222. Further, under the same strain amplitude, the critical values for uncharged samples and hydrogen-charged samples are observed to vary significantly. This indicates that hydrogen charging significantly affects the magnitude of the critical value, along with the LCF life of the samples.

Similarly, References [17,39] employed the standard deviation of axial strain within the RVE and the average value of the first principal stress to characterize microscale deformation inhomogeneity and utilized this as FIP. They obtained the evolution law of FIP with the number of cycles for various metals, as well as the relationship between fatigue life and FIP. Based on this concept, the present study has proposed a new fatigue indicator parameter, incorporating considerations for the effects of hydrogen charging and stress peaks on fatigue life.

### 4.3. Fatigue Life Prediction and Validation

**Figure 12:**
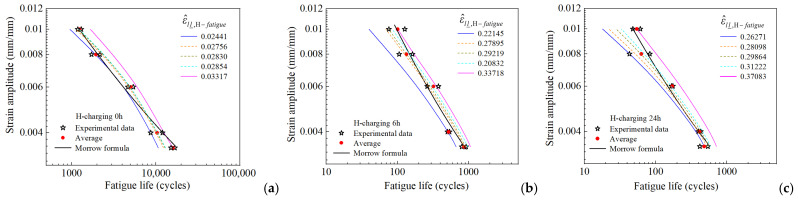
Comparison of strain amplitude-life curves predicted based on the maximum, median, and minimum critical values for the three groups of hydrogen-charged samples with the measured fatigue lives and the Morrow curve: (**a**) 0 h, (**b**) 6 h, (**c**) 24 h hydrogen charging.

To demonstrate the validity of the proposed fatigue criterion (Equation (20)), fatigue life predictions were made for each group of samples based on the corresponding critical values that were obtained previously. The predicted values were then fitted into curves (B-spline fitted curves) to generate a family of predicted fatigue life curves for each of the three groups of hydrogen-charged samples. Additionally, the experimentally measured fatigue lives and the Morrow curve fitted from all the measured fatigue life data (represented by a solid black line) were plotted in the same coordinate system as shown in Figure 12. The figure indicates that the trends of the predicted curve family closely concur with those of the measured fatigue lives, thereby demonstrating the feasibility and effectiveness of the proposed fatigue failure criterion (Equation (20)). For samples with the same hydrogen charging duration under varying strain amplitude conditions, the proposed criterion requires only single-strain-amplitude test data for fatigue life prediction. Conversely, test data from all the strain amplitudes must be utilized for the Morrow curve. The effects of mean stress and hydrogen charging on fatigue life were considered in [32]. Low-cycle fatigue life prediction for hydrogen-charged HRB400 steel was achieved through modification of the Basquin equation. However, calibration using test data from all strain amplitudes is required to determine model parameters before prediction can be achieved using this method. In References [17,18,39], a fatigue life prediction approach was developed using mesoscopic deformation inhomogeneity characterization parameters as FIP. This approach has been applied to various metallic materials. Nevertheless, the influence of internal hydrogen in metallic materials is not accounted for by this method.

**Figure 13:**
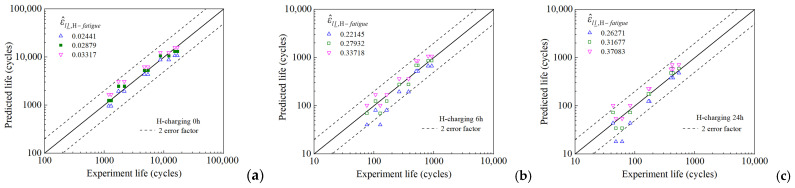
Error assessment between the predicted and measured fatigue lives under strain-controlled cyclic loading conditions based on different critical values: (**a**) 0 h, (**b**) 6 h, (**c**) 24 h hydrogen charging.

The predicted lifetimes for the three groups of hydrogen-charged samples, based on the maximum, median, and minimum critical values, respectively, were plotted as points within a two-fold error band, as shown in Figure 13. The abscissa and ordinate in these figures represent the measured fatigue life and model-predicted fatigue life (based on critical values), respectively. The black solid line indicates the ideal prediction. The dashed black lines indicate the boundaries of the two-fold error band. It can be observed that regardless of whether the critical value is the maximum, median, or minimum value, the predicted lifetimes typically fall within the two-fold error range, with only a few predictions (those with fatigue lives below 10^2^) exceeding this error range. This demonstrates that employing the hydrogen-influenced FIP, ${\hat{\varepsilon }}_{l\underline{l},H}$, helped in effectively predicting the LCF life of hydrogen-pre-charged HRB400 steel samples. Additionally, when the critical value, ${\hat{\varepsilon }}_{l\underline{l},H-fatigue}$, is set to the median value, the predicted fatigue lives are closer to the measured lives, clustering around the solid black line (y = x). This indicates that the prediction accuracy of this method can be significantly enhanced using an increased amount of measured fatigue life data.

In summary, the proposed method used for predicting the LCF life of hydrogen-pre-charged metallic materials was demonstrated to be feasible and effective. This method requires only a single fatigue test at a specific strain amplitude to determine the critical value, ${\hat{\varepsilon }}_{l\underline{l},H-fatigue}$, thereby enabling the prediction of the LCF life for materials under various cyclic loading conditions with the same hydrogen charging duration. Furthermore, utilizing the median value of the critical value for life prediction can enhance the accuracy of the predictions as the amount of fatigue testing and measured life data increases. The proposed method presents the advantages of simplicity and convenience, while significantly improving the efficiency of the fatigue life prediction and reducing resource consumption.

## 5. Conclusions

A new FIP was proposed to predict the LCF life of HRB400 steel under hydrogen influence in this paper. First, the effects of pre-charged hydrogen on the LCF behavior of HRB400 steel were investigated through experimental testing and crystal-plasticity numerical simulations, and the relationship between fatigue life and the evolution of mesoscale deformation inhomogeneity was examined. Second, a new FIP was developed within the framework of CPFE. This FIP is characterized by microscale deformation inhomogeneity and incorporates three internal variables: the hydrogen embrittlement index, coefficient of variation in axial strain, and macroscopic stress ratio. The LCF life of hydrogen-charged HRB400 steel was predicted using the proposed FIP. The primary conclusions drawn from this study are as follows:

(1) Hydrogen charging significantly modifies the cyclic hardening/softening characteristics and cyclic hysteresis behavior of HRB400 steel. It increases the elastic regime within the stabilized hysteresis loop and markedly reduces its LCF life.
(2) The hydrogen-charged HRB400 steel specimens (charged for 0 h, 6 h, and 24 h) all exhibited non-Masing behavior. Their hysteretic response was dependent on the applied strain amplitude, manifesting as distinct elastic ranges under different strain amplitudes.
(3) The cyclically stabilized stress–strain hysteresis loops of HRB400 steel were simulated using the CPFEM. The simulation results demonstrated excellent concurrence with the experimental measurements.
(4) The proposed FIP is effective and feasible for predicting the LCF life of hydrogen-containing metals, with the discrepancies between the predicted and measured results generally within the two-fold error range.
(5) The proposed low-cycle fatigue life prediction method theoretically requires only a single measured fatigue life at a specific strain amplitude to determine the critical value of the FIP, thereby enabling predictions of the LCF life under other cyclic loading conditions. This method presents the advantages of simplicity and efficiency. Furthermore, the prediction accuracy improves with the increase in the amount of measured fatigue life data.

The present method has only been validated for HRB400 steel. Subsequent experiments and simulation establishing defect-fatigue life correlations must be conducted on representative hydrogen-embrittlement-sensitive materials (e.g., high-strength steels and titanium alloys) to assess its universality.

## Figures and Tables

**Table 1:** Electrochemical hydrogen charging conditions for test samples [32].

Group Number	Electrolyte	Current Density	Hydrogen Charging Time
H-charging 0 h	—	—	0 h
H-charging 6 h	0.5 mol/mL H_2_SO_4_ + 0.001 g/mL (NH_2_)_2_CS	5 mA/cm^2^	6 h
H-charging 24 h	0.5 mol/mL H_2_SO_4_ + 0.001 g/mL (NH_2_)_2_CS	5 mA/cm^2^	24 h

**Table 2:** Fatigue strength and fatigue ductility parameters of HRB400 steel with different hydrogen charging times.

Hydrogen-Charged Time	$\sigma_{f}$ (MPa)	*b*	$\varepsilon_{f}$	*c*
0 h	1408.1	−0.128	0.579	−0.558
6 h	1155.5	−0.153	0.271	−0.682
24 h	958.8	−0.135	0.116	−0.602

**Table 3:** Crystal plasticity model parameters for uncharged HRB400 steel at ε_a_ = 0.6%.

$C_{11}$ GPa	$C_{12}$ GPa	$C_{44}$ GPa	$\tau_{0}$ MPa	$\tau_{s}$ MPa	$h_{0}$ MPa	a GPa	c GPa	p *s* ^−1^	$e_{1}$	$e_{2}$	${\dot{\gamma }}_{0}$ *s* ^−1^	q	k
293.4	158.0	67.6	90	94	110	35	0.57	0	0	0	0.001	1	200

**Table 4:** Values of the model parameter $\tau_{s}$ for HRB400 steel under different hydrogen charging durations at various strain amplitudes.

$\mathrm{Strain}\;\mathrm{Amplitude}\;\varepsilon_{a}$	0.35%	0.4%	0.6%	0.8%	1.0%
H-charging 0 h	91	91	94	98	102
H-charging 6 h	99	99	102	105	108
H-charging 24 h	103	103	105	108	111

## Data Availability

The original contributions presented in this study are included in the article. Further inquiries can be directed to the corresponding authors.
